# Perforation grêlique secondaire à un iléus biliaire: à propos d'un cas

**DOI:** 10.11604/pamj.2014.19.270.5463

**Published:** 2014-11-11

**Authors:** Mourad Oussaid, Hicham Elbouhaddouti

**Affiliations:** 1 Service de Chirurgie B, CHU Hassan 2, Fès, Maroc; 2 Service de Chirurgie Viscérale A, CHU Hassan 2, Fès, Maroc

**Keywords:** Iléus biliaire, perforation grêlique, lithiase biliaire, gallstone ileus, small bowel perforation, cholelithiasis

## Image en medicine

L'iléus biliaire est une complication rare de la lithiase biliaire; il est caractérisé par la triade radiologique, syndrome occlusif, aérobilie et localisation ectopique d'un calcul dans le tube digestif. La cause est généralement une fistule cholécysto-duodénale. L'enclavement de calcul au niveau d'une anse qui est généralement la dernière anse iléale peut se compliquer d'une perforation grêlique. Nous rapportons le cas d'un patient de 72 ans, admis pour syndrome subocclusif remontant à une semaine, chez qui l'examen clinique trouve un patient fébrile à 37,8 °C avec une défense péri-ombilicale et épigastrique avec au bilan biologique une CRP à 320 et GB = 8000 elt/mm^3^ et une insuffisance rénale d'allure fonctionnelle. Un ASP a montré des niveaux hydro aériques gêliques et une TDM abdominale - a mis en évidence une aérobilie avec une fistule cholécysto-duodénale associée à pneumopéritoine. L'exploration chirurgicale confirme la présence de la fistule cholécysto-duodénale et objective la présence de deux calcules cholestéroliques au niveau de la lumière intestinale avec une perforation grélique à 1m20 de l'angle de treitz. Le geste chirurgicale a consisté en une résection d'environ 10 cm de grêle emportant la perforation avec extraction de deux gros calcules de 4 et 3 cm de grand axe et transformation des deux bouts grêliques en une stomie à la Bouillie-Wolkman sur le flanc gauche et à un lavage et drainage et nous avons décidé de ne pas libérer la fistule cholécysto-duodénale et de la traiter ultérieurement. Les suites post opératoire immédiates ont été simples.

**Figure 1:**
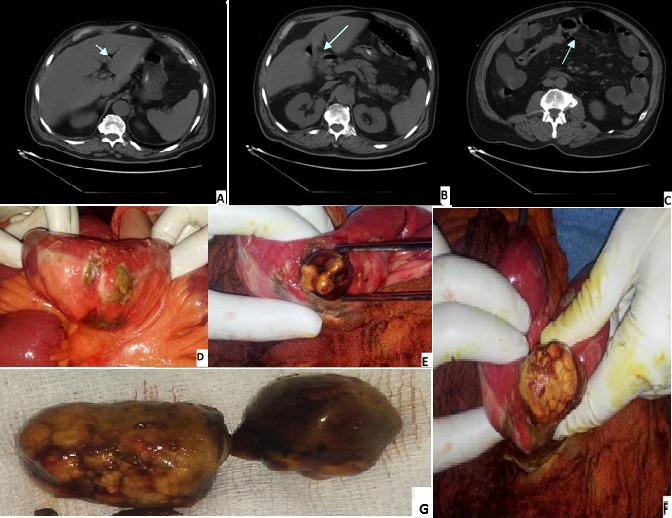
A) images scanographiques et peroperatoires montrant l'aérobilie; B) la fistule cholécystoduodenale; C) le pneumopéritoine; D) la perforation grêlique; E,F) l'extraction des deux gros calculs; G) les deux gros calculs responsable de l’élius biliaire

